# Difference in effects of cigarette smoking or alcohol consumption on serum non-high-density lipoprotein cholesterol levels is related to mitochondrial DNA 5178 C/A polymorphism in middle-aged Japanese men: a cross-sectional study

**DOI:** 10.1186/1880-6805-33-1

**Published:** 2014-01-03

**Authors:** Akatsuki Kokaze, Mamoru Ishikawa, Naomi Matsunaga, Kanae Karita, Masao Yoshida, Tadahiro Ohtsu, Hirotaka Ochiai, Takako Shirasawa, Hinako Nanri, Hiromi Hoshino, Yutaka Takashima

**Affiliations:** 1 Department of Public Health, Showa University School of Medicine, 1-5-8 Hatanodai, 142-8555 Shinagawa-ku, Tokyo, Japan; 2 Department of Public Health, Kyorin University School of Medicine, 6-20-2 Shinkawa, 181-8611 Mitaka-shi, Tokyo, Japan; 3 Mito Red Cross Hospital, 3-12-48 Sannomaru, 310-0011 Mito-shi, Ibaraki, Japan

**Keywords:** Alcohol consumption, Cigarette smoking, Longevity, Mitochondrial DNA polymorphism, Non-HDL cholesterol

## Abstract

**Background:**

Mitochondrial DNA 5178 cytosine/adenosine (Mt5178 C/A) polymorphism is associated with longevity in the Japanese. The purpose of this study is to investigate whether Mt5178 C/A polymorphism modifies the effects of habitual smoking or habitual drinking on serum non-high-density lipoprotein (non-HDL) cholesterol levels in middle-aged Japanese men.

**Methods:**

A total of 394 male subjects (age 53.9 ± 7.9 years; mean ± SD) were selected from among individuals visiting the hospital for regular medical check-ups. After Mt5178 C/A genotyping, a cross-sectional study assessing the joint effects of Mt5178 C/A polymorphism and cigarette smoking or alcohol drinking on serum non-HDL cholesterol levels was conducted. High levels of serum non-HDL cholesterol were defined as serum non-HDL cholesterol levels ≥160 mg/dl or ≥190 mg/dl.

**Results:**

For men with Mt5178A, cigarette smoking may increase serum non-HDL cholesterol levels (*P* for trend < 0.001), as well as the risk of high levels of non-HDL cholesterol (serum non-HDL cholesterol levels ≥160 mg/dl, *P* for trend < 0.001; serum non-HDL cholesterol levels ≥190 mg/dl, *P* for trend = 0.004). On the other hand, for men with Mt5178C, after adjusting for age and body mass index, alcohol consumption may decrease serum non-HDL cholesterol levels (*P* for trend = 0.043) and the risk of high levels of non-HDL cholesterol (serum non-HDL cholesterol level ≥160 mg/dl, *P* for trend = 0.005).

**Conclusions:**

These gene-environment interactions on serum non-HDL cholesterol levels may contribute to the establishment of individualized prevention of the risk of high levels of serum non-HDL cholesterol.

## Background

Non-high-density lipoprotein (non-HDL) cholesterol, which includes the cholesterol carried by apolipoprotein B-containing particles, has a clear pathophysiologic link to the development of atherosclerosis, and outperforms other lipid measures in predicting both subclinical atherosclerosis and cardiovascular disease [1]. Orakzai and colleagues also reported that non-HDL cholesterol is more strongly associated with subclinical atherosclerosis than all other routine lipid measures, and proposed that non-HDL cholesterol is a crucial treatment target in primary prevention of coronary heart disease [2].

It is important for primary prevention to detect modifiable factors influencing serum non-HDL cholesterol levels. Large-scale population-based cross-sectional studies have indicated that serum non-HDL cholesterol levels are strongly influenced by cigarette smoking [3] and alcohol consumption [3,4]. Several gene-environment interactions on serum non-HDL cholesterol levels have been published [5-7]. Vogel and colleagues reported the combined effects of cyclooxygenase-2 (COX-2) polymorphism and alcohol consumption on serum non-HDL cholesterol levels [5]. Therefore, even in the establishment of primary prevention for high levels of non-HDL cholesterol, genetic information deserves consideration.

In population genetics, mitochondrial DNA sequences are utilized for climatic adaptation research [8,9]. Mitochondrial DNA haplogroups A, C, D and X reportedly experience much greater cold stress than haplogroup B or L [8]. In physiological anthropology, Nishimura and colleagues reported that seasonal cold acclimatization in haplogroup D individuals relies on more efficient metabolism, while that in haplogroup non-D individuals relies more on insulation [10]. Mitochondrial DNA 5178 cytosine/adenine (Mt5178 C/A) polymorphism is also recognized as NADH dehydrogenase subunit-2 237 leucine/methionine (ND2-237 Leu/Met) polymorphism. Mt5178A is a genetic marker of haplogroup D. The frequency of the Mt5178A genotype is significantly higher in Japanese centenarians than in the general population and, consequently, Mt5178 C/A polymorphism is associated with longevity in the Japanese [11]. Although Mt5178C genotype is reportedly related to performance in elite endurance runners [12], Japanese individuals with Mt5178C are more susceptible to lifestyle-related atherosclerotic diseases, such as myocardial infarction [13,14] and cerebrovascular disorders [15], than those with Mt5178A. Moreover, this polymorphism subclinically influences the effects of habitual smoking and habitual drinking on serum lipid levels [16], and reportedly modifies their effects on the risk of dyslipidemia [17,18]. However, to our knowledge, there have been no reports focusing on the combined effects of Mt5178 C/A polymorphism and cigarette smoking or alcohol consumption on serum non-HDL cholesterol levels.

This study aims to investigate whether longevity-associated Mt5178 C/A polymorphism influences the effects of habitual drinking or habitual smoking on serum non-HDL cholesterol levels or the risk of high levels of serum non-HDL cholesterol in middle-aged Japanese male subjects.

## Subjects and methods

### Study subjects

Participants were recruited from among individuals visiting the Mito Red Cross Hospital for regular medical check-ups between August 1999 and August 2000. This study was conducted in accordance with the Declaration of Helsinki and was approved by the Ethics Committee of Kyorin University School of Medicine. Written informed consent was obtained from 602 volunteers before participation. Because the number of women was insufficient for classification into groups based on Mt5178 C/A genotype and smoking status, women were excluded. Patients taking antihyperlipidemic medication were excluded, and diabetic patients were also excluded because of the higher prevalence of dyslipidemia in diabetic patients when compared with non-diabetic patients [19]. Thus, 406 men were enrolled in the study. Twelve individuals with unclear data were also excluded. Therefore, subjects comprised 394 Japanese men (age 53.9 ± 7.9 years; mean ± SD).

### Clinical characteristics of subjects

Determination of blood chemical and physical data was conducted as described previously [20]. Serum non-HDL cholesterol levels were calculated by subtracting serum HDL cholesterol levels from serum total cholesterol levels. According to the National Cholesterol Education Program Adult Treatment Panel III (ATP III) [21], serum low-density lipoprotein (LDL) cholesterol <100 mg/dl is optimal, 100 to 129 mg/dl is near optimal, 130 to 159 mg/dl is borderline high, 160 to 189 mg/dl is high, and ≥190 mg/dl is very high. ATP III recommends that the goal for serum non-HDL cholesterol be 30 mg/dl higher than the serum LDL cholesterol goal [21]. Therefore, high levels of serum non-HDL cholesterol were preventively or clinically defined as serum non-HDL cholesterol levels ≥160 mg/dl or ≥190 mg/dl. Body mass index (BMI) was defined as the ratio of subject weight (kg) to the square of subject height (m). A survey of cigarette smoking, alcohol consumption and coffee consumption was performed by means of questionnaire. Smoking status was classified based on the number of cigarettes smoked per day (never- or ex-smokers; 1 to 20 cigarettes smoked per day; and >20 cigarettes smoked per day). Alcohol consumption was classified based on drinking frequency (daily drinkers; occasional drinkers, which include those who drink several times per week or per month; and non- or ex-drinkers). Coffee consumption was classified based on number of cups of coffee per day (≤1 cup per day; 2 to 3 cups per day; and ≥4 cups per day). For use of antihypertensive medication, subjects were classified as taking no drug treatment or taking medicine.

### Genotyping

DNA was extracted from white blood cells using the DNA Extractor WB kit (Wako Pure Chemical Industries, Osaka, Japan). Mt5178 C/A polymorphism was detected by polymerase chain reaction (PCR) and digestion with *Alu*I restriction enzyme. The sequence of primers was: forward 5′-CTTAGCATACTCCTCAATTACCC-3′, reverse 5′-GTGAATTCTTCGATAATGGCCCA-3′. PCR was performed with 50 ng genomic DNA in buffer containing 0.2 μmol/l each primer, 1.25 mmol/l dNTPs, 1.5 mmol/l MgCl_2_, and 1 U Taq DNA polymerase (Takara Bio Inc., Shiga, Japan). After an initial denaturation at 94°C for 5 minutes, PCR was conducted through 40 cycles as follows: denaturation at 94°C for 30 seconds, annealing at 60°C for 60 seconds and polymerase extension at 72°C for 90 seconds. After cycling, a final extension at 72°C for 10 minutes was performed. PCR products were digested with the *Alu*I restriction enzyme (Nippon Gene, Tokyo, Japan) at 37°C overnight, followed by electrophoresis in 1.5% agarose gels and staining with ethidium bromide for visualization under ultraviolet light. The absence of an *Alu*I site was designated as Mt5178A, and the presence of this restriction site was designated as Mt5178C.

### Statistical analyses

Statistical analyses were performed using SAS statistical software, version 9.2 for Windows (SAS Institute, Inc., Cary, North Carolina). Multiple logistic regression analysis was used to calculate odds ratios (ORs) for high levels of serum non-HDL cholesterol. For multiple logistic regression analysis and analysis of covariance, habitual alcohol consumption (non- or ex- drinkers = 0, occasional drinkers = 1, daily drinkers = 2), coffee consumption (≤1 cup per day = 1, 2 to 3 cups per day = 2, ≥4 cups per day = 3) and antihypertensive medication use (no use of antihypertensive = 0, use of antihypertensive = 1) were numerically coded. Differences with *P* values of less than 0.05 were considered to be statistically significant.

## Results

No significant differences in biophysical or biochemical characteristics were observed between the Mt5178C and Mt5178A genotypes (Table 1). No significant differences in serum non-HDL cholesterol levels were observed between the Mt5178 C/A genotypes. No significant differences in smoking status or alcohol consumption status were observed between the Mt5178 C/A genotypes.

**Table 1:** Clinical characteristics of study subjects by Mt5178 C/A genotype

	**Mt5178C**	**Mt5178A**	***P *** **value**
	**N = 239**	**N = 155**	
Age (years)	54.3 ± 7.8	53.2 ± 7.8	0.171
Body mass index (kg/m^2^)	23.3 ± 2.8	23.5 ± 2.6	0.461
Serum total cholesterol (mg/dl)	203.5 ± 34.3	202.1 ± 31.8	0.672
Serum HDL cholesterol (mg/dl)	54.6 ± 13.6	56.3 ± 16.2	0.269
Serum non-HDL cholesterol (mg/dl)	148.9 ± 36.3	145.8 ± 34.6	0.395
Serum LDL cholesterol (mg/dl)	121.6 ± 34.8	117.9 ± 30.5	0.281
Serum triglyceride (mg/dl)	136.7 ± 91.1	139.5 ± 90.8	0.766
Systolic blood pressure (mmHg)	125.8 ± 15.9	125.7 ± 14.1	0.940
Diastolic blood pressure (mmHg)	74.0 ± 10.7	73.8 ± 9.1	0.823
Fasting plasma glucose (mg/dl)	97.2 ± 9.4	97.6 ± 9.7	0.672
Smoking status (never- or ex-/1-20 cigarettes per day/>20 cigarettes per day) (%)	59.4/29.7/10.9	59.4/25.8/14.8	0.429
Alcohol consumption (daily/occasionally/non- or ex-) (%)	46.4/35.2/18.4	47.7/38.7/13.6	0.426
Coffee consumption (≤1 cup per day/2-3 cups per day/≥4 cups per day) (%)	61.1/29.7/9.2	55.5/32.9/11.6	0.510
Antihypertensive (%)	18.8	12.9	0.122

Age, body mass index, serum total cholesterol levels, serum HDL cholesterol levels, serum non-HDL cholesterol levels, serum LDL cholesterol levels, serum triglyceride levels, systolic blood pressure, diastolic blood pressure and fasting plasma glucose levels are given as means ± SD. For smoking status, alcohol consumption, coffee consumption and antihypertensive use, *P* values were calculated by chi-squared test. All *P* values depict significance of differences between mitochondrial DNA 5178 cytosine (Mt5178C) and mitochondrial DNA 5178 adenosine (Mt5178A). HDL, high-density lipoprotein; LDL, low-density lipoprotein.

For Mt5178A genotypic men, the number of cigarettes smoked per day was positively and significantly associated with serum non-HDL cholesterol levels (*P* for trend < 0.001) (Table 2). After adjustment for age and BMI, or for age, BMI, alcohol consumption, coffee consumption and use of antihypertensive medication, the number of cigarettes smoked per day was also positively and significantly associated with serum non-HDL cholesterol levels (adjusted *P* for trend < 0.001, and adjusted *P* for trend < 0.001, respectively). Serum non-HDL cholesterol levels were significantly higher in those who smoked >20 cigarettes per day than in never- or ex-smokers (*P* < 0.001). After adjustment for age and BMI, or for age, BMI, alcohol consumption, coffee consumption and use of antihypertensive medication, serum non-HDL cholesterol levels were significantly higher in those who smoked >20 cigarettes per day than in never- or ex-smokers (*P* = 0.001 and *P* = 0.002, respectively). Serum non-HDL cholesterol levels were significantly higher in those who consumed >20 cigarettes per day than in those who consumed 1 to 20 cigarettes per day (*P* = 0.023). After adjustment, a significant difference was not observed. However, for Mt5178C genotypic men, no significant associations between cigarette smoking and serum non-HDL cholesterol levels were observed.

**Table 2:** Serum non-HDL cholesterol levels classified by smoking status and Mt5178 C/A genotype

	**Smoking status**			***P *** **for trend**
	**Never- or ex-smokers**	**1-20 cigarettes smoked per day**	**>20 cigarettes smoked per day**	
Mt5178C	N = 142	N = 70	N = 27	
non-HDL cholesterol	149.2 ± 3.0	144.7 ± 4.3	158.6 ± 7.0	*P* = 0.557
non-HDL cholesterol^†^	148.8 ± 3.0	145.4 ± 4.3	160.5 ± 7.0	*P* = 0.333
non-HDL cholesterol^‡^	151.8 ± 4.6	146.8 ± 5.2	162.3 ± 7.7	*P* = 0.500
Mt5178A	N = 92	N = 40	N = 23	
non-HDL cholesterol	139.9 ± 3.5	145.8 ± 5.3	169.3 ± 6.9**^,§^	*P* < 0.001
non-HDL cholesterol^†^	139.0 ± 3.4	149.4 ± 5.2	166.7 ± 6.7*	*P* < 0.001
non-HDL cholesterol^‡^	141.2 ± 5.4	150.1 ± 6.1	168.0 ± 8.1*	*P* < 0.001

^†^non-HDL cholesterol levels are given as least-square mean ± SE adjusted for age and body mass index; ^‡^non-HDL cholesterol levels are given as least-square mean ± SE adjusted for age, body mass index, alcohol consumption, coffee consumption and antihypertensive medication use. Bonferroni correction for multiple comparisons was applied. *P < 0.005 vs never- or ex-smokers, **P < 0.001 vs never- or ex-smokers, ^§^*P* < 0.001 vs current smokers who smoked 1 to 20 cigarettes per day. HDL, high-density lipoprotein; Mt5178C, mitochondrial DNA 5178 cytosine; Mt5178A, mitochondrial DNA 5178 adenosine.

In the case of high levels of serum non-HDL cholesterol defined as serum non-HDL cholesterol levels ≥160 mg/dl, for subjects with Mt5178A the risk of high levels of serum non-HDL cholesterol may depend on daily number of cigarettes smoked (*P* for trend < 0.001) (Table 3). After adjustment, the positive association between increasing daily number of cigarettes smoked and the risk of high levels of serum non-HDL cholesterol remained significant. The crude OR for high levels of serum non-HDL cholesterol was significantly higher in subjects who smoked 1 to 20 cigarettes per day or in those who smoked >20 cigarettes per day than in never- or ex-smokers (OR = 2.254, 95% confidence interval (CI) = 1.015 to 5.007, *P* = 0.046, and OR = 6.339, 95% CI = 2.364 to 17.00, *P* < 0.001, respectively). After adjustment for age and BMI, or for age, BMI, habitual alcohol consumption, coffee consumption and use of antihypertensive medication, a significant OR remained. In the case of high levels of serum non-HDL cholesterol defined as serum non-HDL cholesterol levels ≥190 mg/dl, the risk of high levels of serum non-HDL cholesterol may also depend on daily number of cigarettes smoked (*P* for trend = 0.004). After adjustment, the positive association between increasing daily number of cigarettes smoked and the risk of high levels of serum non-HDL cholesterol also remained significant. The crude OR for high levels of serum non-HDL cholesterol was significantly higher in subjects who consumed >20 cigarettes per day than in never- or ex-smokers (OR = 6.271, 95% CI = 1.862 to 21.11, *P* = 0.003). After adjusting for age and BMI, or for age, BMI, habitual alcohol consumption, coffee consumption and use of antihypertensive medication, a significant OR remained. On the other hand, for neither definition of high levels of serum non-HDL cholesterol, the association between Mt5178C genotype and the risk of high levels of serum non-HDL cholesterol was dependent on the daily number of cigarettes smoked.

**Table 3:** Odds ratios and 95% confidence intervals for high levels of serum non-HDL cholesterol by Mt5178 C/A genotype and smoking status

**Genotype and number of cigarettes smoked daily**	**Frequency**		**OR (95% CI)**	**Adjusted OR**^**† **^**(95% CI)**	**Adjusted OR**^**‡ **^**(95% CI)**
	**Normal levels of serum non-HDL cholesterol**	**High levels of serum non-HDL cholesterol**			
	non-HDL cholesterol <160 mg/dl	non-HDL cholesterol ≥160 mg/dl			
Mt5178C					
0 (never- or ex-smokers)	95 (66.9)	47 (33.1)	1 (reference)	1 (reference)	1 (reference)
1-20	46 (65.7)	24 (34.3)	1.055 (0.576-1.931)	1.079 (0.577-2.016)	0.955 (0.491-1.857)
>20	16 (59.3)	11 (40.7)	1.390 (0.598-3.230)	1.436 (0.591-3.492)	1.498 (0.596-3.765)
			*P* for trend = 0.495	*P* for trend = 0.403	*P* for trend = 0.619
Mt5178A					
0 (never- or ex-smokers)	71 (77.2)	21 (22.8)	1 (reference)	1 (reference)	1 (reference)
1-20	24 (60.0)	16 (40.0)	2.254 (1.015-5.007)*	3.035 (1.269-7.259)*	2.580 (1.027-6.481)*
>20	8 (34.8)	15 (65.2)	6.339 (2.364-17.00)***	6.191 (2.258-16.97)***	5.681 (2.022-15.96)**
			*P* for trend < 0.001	*P* for trend < 0.001	*P* for trend < 0.001
	non-HDL cholesterol <190 mg/dl	non-HDL cholesterol ≥190 mg/dl			
Mt5178C					
0 (never- or ex-smokers)	126 (88.7)	16 (11.3)	1 (reference)	1 (reference)	1 (reference)
1-20	62 (88.6)	8 (11.4)	1.016 (0.413-2.503)	1.071 (0.427-2.686)	0.890 (0.334-2.376)
>20	22 (81.5)	5 (18.5)	1.790 (0.595-5.385)	1.943 (0.6.5-6.244)	2.210 (0.654-7.469)
			*P* for trend = 0.397	*P* for trend = 0.313	*P* for trend = 0.368
Mt5178A					
0 (never- or ex-smokers)	86 (93.5)	6 (6.5)	1 (reference)	1 (reference)	1 (reference)
1-20	35 (87.5)	2 (12.5)	2.048 (0.587-7.148)	2.420 (0.655-8.944)	2.815 (0.692-11.45)
>20	16 (69.6)	7 (30.4)	6.271 (1.862-21.11)**	6.081 (1.772-20.86)**	6.315 (1.715-23.25)*
			*P* for trend = 0.004	*P* for trend = 0.006	*P* for trend = 0.007

^†^OR adjusted for age and body mass index. ^‡^OR adjusted for age, body mass index, habitual alcohol consumption, coffee consumption and antihypertensive medication. *P < 0.05, **P < 0.005, ***P < 0.001. CI, confidence interval; HDL, high-density lipoprotein; Mt5178C, mitochondrial DNA 5178 cytosine; Mt5178A, mitochondrial DNA 5178 adenosine; OR, odds ratio.

For Mt5178C genotypic men, alcohol consumption was negatively associated with serum non-HDL cholesterol levels (Table 4). After adjustment for age and BMI, or for age, BMI, cigarette smoking, coffee consumption and use of antihypertensive medication, alcohol consumption was negatively and significantly associated with serum non-HDL cholesterol levels (adjusted *P* for trend = 0.043 and adjusted *P* for trend = 0.040, respectively). After adjustment for age and BMI, serum non-HDL cholesterol levels were significantly lower in occasional drinkers or in daily drinkers than in non- or ex-drinkers (*P* = 0.049 and *P* = 0.047, respectively). Moreover, after adjustment for age and BMI or for age, BMI, cigarette smoking, coffee consumption and use of antihypertensive medication, serum non-HDL cholesterol levels were significantly lower in occasional drinkers or in daily drinkers than in non- or ex-drinkers (*P* = 0.048 and *P* = 0.044, respectively). On the other hand, for Mt5178A genotypic men, no significant associations between alcohol consumption and serum non-HDL cholesterol levels were observed.

**Table 4:** Serum non-HDL cholesterol levels classified by drinking status and Mt5178 C/A genotype

	**Drinking status**			***P *** **for trend**
	**Non- or ex-drinkers**	**Occasional drinkers**	**Daily drinkers**	
Mt5178C	N = 44	N = 84	N = 111	
non-HDL cholesterol	159.8 ± 5.4	146.2 ± 3.9	146.7 ± 3.4	*P* = 0.088
non-HDL cholesterol^†^	161.8 ± 5.4	145.7 ± 3.9*	146.3 ± 3.4*	*P* = 0.043
non-HDL cholesterol^‡^	164.5 ± 6.7	148.0 ± 5.5*	148.4 ± 4.6*	*P* = 0.040
Mt5178A	N = 21	N = 60	N = 74	
non-HDL cholesterol	151.2 ± 7.6	147.9 ± 4.5	142.6 ± 4.0	*P* = 0.245
non-HDL cholesterol^†^	153.7 ± 7.4	147.6 ± 4.3	142.1 ± 3.9	*P* = 0.140
non-HDL cholesterol^‡^	159.7 ± 8.3	152.3 ± 5.7	147.3 ± 5.3	*P* = 0.114

^†^non-HDL cholesterol levels are given as least-square mean ± SE adjusted for age and body mass index; ^‡^non-HDL cholesterol levels are given as least-square mean ± SE adjusted for age, body mass index, cigarette smoking, coffee consumption and antihypertensive medication use. Bonferroni correction for multiple comparisons was applied. *P < 0.05 vs non- or ex-drinkers. HDL, high-density lipoprotein; Mt5178C, mitochondrial DNA 5178 cytosine; Mt5178A, mitochondrial DNA 5178 adenosine.

In the case of high levels of serum non-HDL cholesterol defined as serum non-HDL cholesterol levels ≥160 mg/dl, for subjects with Mt5178C the risk of high levels of serum non-HDL cholesterol may depend on alcohol consumption (*P* for trend = 0.012) (Table 5). After adjustment, the negative association between increasing frequency of alcohol consumption and the risk of high levels of serum non-HDL cholesterol remained significant. The crude OR for high levels of serum non-HDL cholesterol was significantly lower in daily drinkers than in non- or ex-drinkers (OR = 0.388, 95% CI = 0.188 to 0.798, *P* = 0.010). After adjustment for age and BMI, or for age, BMI, cigarette smoking, coffee consumption and use of antihypertensive medication, a significant OR remained. After adjustment for age and BMI only, the OR for high levels of serum non-HDL cholesterol was significantly lower in occasional drinkers than in non- or ex-drinkers (OR = 0.430, 95% CI = 0.194 to 0.953, *P* = 0.038). However, in the case of high levels of serum non-HDL cholesterol defined as serum non-HDL cholesterol levels ≥190 mg/dl, the association between Mt5178C genotype and the risk of high levels of serum non-HDL cholesterol was not dependent on alcohol consumption. On the other hand, for neither definition of high levels of serum non-HDL cholesterol, the association between Mt5178A genotype and the risk of high levels of serum non-HDL cholesterol was dependent on alcohol consumption.

**Table 5:** Odds ratios and 95% confidence intervals for high levels of serum non-HDL cholesterol by Mt5178 C/A genotype and drinking status

**Genotype and alcohol consumption**	**Frequency**		**OR (95% CI)**	**Adjusted OR**^**† **^**(95% CI)**	**Adjusted OR**^**‡ **^**(95% CI)**
	**Normal levels of serum non-HDL cholesterol**	**High levels of serum non-HDL cholesterol**			
	non-HDL cholesterol <160 mg/dl	non-HDL cholesterol ≥160 mg/dl			
Mt5178C					
Non- or ex-drinkers	22 (50.0)	22 (50.0)	1 (reference)	1 (reference)	1 (reference)
Occasional drinkers	55 (65.5)	29 (34.5)	0.527 (0.251-1.108)	0.430 (0.194-0.953)*	0.439 (0.190-1.016)
Daily drinkers	80 (72.1)	31 (27.9)	0.388 (0.188-0.798)*	0.308 (0.142-0.665)**	0.312 (0.139-0.698)**
			*P* for trend = 0.012	*P* for trend = 0.005	*P* for trend = 0.005
Mt5178A					
Non- or ex-drinkers	17 (81.0)	4 (19.0)	1 (reference)	1 (reference)	1 (reference)
Occasional drinkers	38 (63.3)	22 (36.7)	2.461 (0.734-8.244)	2.491 (0.720-8.619)	2.499 (0.649-9.618)
Daily drinkers	48 (64.9)	26 (35.1)	2.302 (0.701-7.561)	2.022 (0.595-6.875)	2.114 (0.583-7.665)
			*P* for trend = 0.310	*P* for trend = 0.385	*P* for trend = 0.348
	non-HDL cholesterol <190 mg/dl	non-HDL cholesterol ≥190 mg/dl			
Mt5178C					
Non- or ex-drinkers	36 (81.5)	8 (18.2)	1 (reference)	1 (reference)	1 (reference)
Occasional drinkers	74 (88.1)	10 (11.9)	0.608 (0.221-1.672)	0.627 (0.221-1.780)	0.858 (0.279-2.640)
Daily drinkers	100 (90.1)	11 (9.9)	0.495 (0.184-1.328)	0.408 (0.146-1.139)	0.330 (0.108-1.003)
			*P* for trend = 0.181	*P* for trend = 0.140	*P* for trend = 0.123
Mt5178A					
Non- or ex-drinkers	19 (90.5)	2 (9.5)	1 (reference)	1 (reference)	1 (reference)
Occasional drinkers	54 (90.0)	6 (10.0)	1.056 (0.196-5.684)	1.014 (0.176-5.853)	0.938 (0.154-5.714)
Daily drinkers	64 (86.5)	10 (13.5)	1.484 (0.299-7.368)	1.190 (0.230-6.156)	1.325 (0.219-7.996)
			*P* for trend = 0.513	*P* for trend = 0.605	*P* for trend = 0.560

^†^OR adjusted for age and body mass index. ^‡^OR adjusted for age, body mass index, cigarette smoking, coffee consumption and antihypertensive medication. *P < 0.05, **P < 0.005. CI, confidence interval; HDL, high-density lipoprotein; Mt5178C, mitochondrial DNA 5178 cytosine; Mt5178A, mitochondrial DNA 5178 adenosine; OR, odds ratio.

## Discussion

In the present study, we observed that Mt5178 C/A polymorphism may influence the effects of habitual smoking or habitual drinking on serum non-HDL cholesterol levels and the risk of high levels of non-HDL cholesterol in middle-aged male Japanese subjects. Cigarette smoking may increase serum non-HDL cholesterol levels and the risk of high levels of non-HDL cholesterol for Mt5178A genotypic men. However, the effects of cigarette smoking seem to be equivocal on both of these in Mt5178C genotypic men, while alcohol consumption may decrease both serum non-HDL cholesterol levels and the risk of high levels of non-HDL cholesterol in Mt5178C genotypic men. These findings are consistent with our previous investigations reporting the joint effects of Mt5178 C/A polymorphism and habitual drinking or habitual smoking on the risk of dyslipidemia [17,18].

Wakabayashi and Groschner reported that even light alcohol consumption is sufficient to lower serum non-HDL cholesterol levels and that this effect of drinking on serum non-HDL cholesterol is altered by sex [4]. Moreover, they reported that smoking is associated with high non-HDL cholesterol in non-drinkers, that alcohol consumption is associated with low non-HDL cholesterol in non-smokers, and that these associations are modified by age [3]. Here, we showed that a genetic factor also influences the effects of smoking or drinking on serum non-HDL cholesterol levels.

Genotyping of Mt5178 C/A appears to establish personalized prevention of dyslipidemia. Abstaining from coffee intake may be recommended for Mt5178A genotypic men to maximize the genetic advantage toward lower risk of hyper-LDL cholesterolemia [22]. Smoking cessation may be also advocated to lower the risk of hyper-LDL cholesterolemia [18], that of hypertriglyceridemia [18] and that of high levels of serum non-HDL cholesterol. Daily alcohol consumption may reduce the risk of hyper-LDL cholesterolemia [17] and the risk of high levels of serum non-HDL cholesterol for men with Mt5178C. Smoking cessation may be also urged for such individuals to lower the risk of hypo-HDL cholesterolemia [18].

The mechanisms underlying the combined effects of Mt5178 C/A polymorphism and lifestyle, namely habitual smoking or habitual drinking, on serum non-HDL cholesterol levels have not been clarified. Clinical epidemiological studies have indicated that individuals with the Mt5178A genotype are more resistant to atherosclerotic diseases than those with the Mt5178C genotype [13-15]. The biochemical attributes of ND2-237Met are thought to be responsible for these antiatherogenic benefits. NADH dehydrogenase is considered to be the major physiologically and pathologically relevant reactive oxygen species (ROS)-generating site in mitochondria, and NADH dehydrogenase itself is sensitive to inhibition by ROS [23]. Mitochondrial DNA is also exposed to damage by ROS [23]. Increased mitochondrial ROS production, mitochondrial dysfunction and mitochondrial DNA mutation are related to atherosclerotic diseases [23]. Considering that methionine residues act as endogenous antioxidants [24], ND2-237Met may protect NADH dehydrogenase itself and mitochondrial DNA from ROS. Moreover, extrapolation of results using experimental mice [25] to humans assumes that ND2-237Met also suppresses ROS production. Cigarette smoke contains high concentrations of ROS, and is thought to activate endogenous sources of ROS [26]. Ethanol metabolism is directly involved in the production of ROS [27]. There may be biochemical differences in the protection against cigarette smoking- or alcohol consumption-induced ROS or the reduction of cigarette smoking- or alcohol consumption-induced ROS generation between ND2-237Leu and ND2-237Met. These deduced differences are conjectured to bring about joint effects of Mt5178 C/A polymorphism and cigarette smoking or alcohol consumption on serum non-HDL cholesterol levels or the risk of high levels of serum non-HDL cholesterol. However, in order to determine the mechanisms responsible for the combined effects of ND2-237 Leu/Met genotypes and lifestyles on serum non-HDL cholesterol levels or the risk of high levels of serum non-HDL cholesterol, further biochemical investigations are required.

Several genetic polymorphisms are reportedly associated with the concentrations of serum non-HDL cholesterol [5-7,28-30]. Apolipoprotein E polymorphism influences serum non-HDL cholesterol levels [28,29]. Although in postmenopausal women, human SA gene polymorphisms modulate serum non-HDL cholesterol levels [30]. Fatty acid desaturase 1 polymorphism interacts with dietary ω-3 polyunsaturated fatty acid intake [6] or dietary α-linolenic acid [7] to affect serum non-HDL cholesterol levels, and COX-2 8473 thymine/cytosine polymorphism modulates the effects of alcohol consumption on serum non-HDL cholesterol levels [5]. Therefore, whether there are gene-gene interactions or gene-gene-environment interactions on serum non-HDL cholesterol levels warrants further investigation.

Several potential limitations should be considered. First, sample size was too small to validate the gene-environment interaction. Second, we cannot rule out selection bias due to our recruiting of subjects from those visiting the hospital for regular medical check-ups. Third, a cross-sectional study cannot establish a valid causality. Fourth, we cannot rule out the possibility of false-positive joint effects of Mt5178 C/A polymorphism and habitual smoking or habitual drinking on serum non-HDL cholesterol levels. Namely, Mt5178 C/A polymorphism may only reflect the population structure, and another genetic factor inherited with Mt5178 C/A genotype may interact with cigarette smoking or alcohol consumption on serum non-HDL cholesterol levels. Therefore, in order to overcome these limitations, a large-scale population-based follow-up genome-wide association study is necessary. Fifth, this study was an evaluation of habitual drinking based on the frequency of alcohol consumption. Although we have used this evaluation in previous studies [16,17,31,32], validation of a combined effect between Mt5178 C/A polymorphism and volume of alcohol intake on serum non-HDL cholesterol levels will be necessary. Finally, to determine the joint effects of genetic polymorphisms and nutrient factors on serum non-HDL cholesterol levels [6,7], a dietary survey will also be required.

## Conclusions

Mt5178 C/A polymorphism may modify the effects of habitual smoking or habitual drinking on serum non-HDL cholesterol levels and the risk of high levels of non-HDL cholesterol. Our findings suggest novel gene-environment interactions on serum non-HDL cholesterol levels, which are considered to be a risk marker for coronary heart disease [1,2], and may contribute to the establishment of individualized prevention of high levels of serum non-HDL cholesterol and, thereby, protection against cardiovascular diseases. Moreover, considering the advantages of studying gene-environment interactions across heterogeneous groups with diverse lifestyles [33], this investigation may provide insights in anthropological fields.

## Abbreviations

ATP III: Adult treatment panel III; BMI: Body mass index; CI: Confidence interval; COX-2: cyclooxygenase 2; HDL: High-density lipoprotein; LDL: Low-density lipoprotein; Mt5178 C/A: mitochondrial DNA 5178 cytosine/adenosine; ND2-237 Leu/Met: NADH dehydrogenase subunit-2 237 leucine/methionine; OR: Odds ratio; PCR: Polymerase chain reaction; ROS: Reactive oxygen species.

## Competing interests

The authors declare that they have no competing interests.

## Authors’ contributions

AK designed the study, carried out the epidemiological survey, carried out genotyping, analyzed the data, and drafted the manuscript; MI collected the samples; NM assisted with genotyping; KK and MY carried out the epidemiological survey; TO, HO, TS, HN and HH assisted in data analysis and helped with interpreting the results; YT designed the study and carried out the epidemiological survey. All authors have read and approved the final manuscript.
